# Human Serum Albumin‐Coated ^10^B Enriched Carbon Dots as Targeted “Pilot Light” for Boron Neutron Capture Therapy

**DOI:** 10.1002/advs.202406577

**Published:** 2024-09-26

**Authors:** Tianyuan Zhong, Yongjin Yang, Miao Pang, Yong Pan, Shiwei Jing, Yanxin Qi, Yubin Huang

**Affiliations:** ^1^ Faculty of Chemistry Northeast Normal University Changchun 130024 China; ^2^ Department of Urology The Second Hospital & Clinical Medical School Lanzhou University Lanzhou Gansu 730000 China; ^3^ Gansu Province Clinical Research Center for Urinary System Disease Lanzhou Gansu 730000 China; ^4^ School of Physics Northeast Normal University Changchun 130024 China

**Keywords:** boron‐containing carbon dots, boron neutron capture therapy, human serum albumin, tumor targeting

## Abstract

Boron neutron capture therapy (BNCT) is a physiologically focused radiation therapy that relies on nuclear capture and fission processes. BNCT is regarded as one of the most promising treatments due to its excellent accuracy, short duration of therapy, and low side effects. The creation of novel boron medicines with high selectivity, ease of delivery, and high boron‐effective load is a current research topic. Herein, boron‐containing carbon dots (BCDs) and their human serum albumin (HSA) complexes (BCDs‐HSA) are designed and synthesized as boron‐containing drugs for BNCT. BCDs (^10^B: 7.1 wt%) and BCDs‐HSA exhibited excitation‐independent orange fluorescent emission which supported the use of fluorescence imaging for tracking ^10^B in vivo. The introduction of HSA enabled BCDs‐HSA to exhibit good biocompatibility and increased tumor accumulation. The active and passive targeting abilities of BCDs‐HSA are explored in detail. Subcutaneous RM‐1 tumors and B16‐F10 tumors both significantly decrease with BNCT, which consists of injecting BCDs‐HSA and then irradiating the area with neutrons. In short, this study provides a novel strategy for the delivery of boron and may broaden the perspectives for the design of boron‐containing carbon dots nanomedicine for BNCT.

## Graphical abstract

Herein, the complexes (BCDs‐HSA) of human serum albumin (HSA) and BCDs are designed and synthesized as ^10^B drugs for Boron neutron capture therapy (BNCT). This work focuses on solving the problems of ^10^B content, tumor targeting, and traceability encountered in the practical application of ^10^B drugs in BNCT, providing a novel strategy for the delivery of ^10^B.

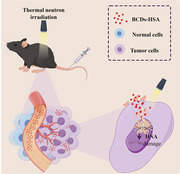

## 1 Introduction

Radiotherapy (RT) is an effective method for the treatment of malignant tumors.^[^ ^1^ ^]^ More than 50% of cancer patients require radiotherapy during treatment.^[^ ^2^ ^]^ Radiation therapy has advanced from conventional non‐selective X‐ and γ‐ray line radiation to targeted radiation as a result of advancements in medical technology.^[^ ^3^ ^]^ Boron Neutron Capture Therapy (BNCT) is a biologically targeted radiation therapy modality, which has become one of the best means of precision radiotherapy for malignant tumors due to its advantages of high efficiency in killing tumor cells at the cellular scale, high accuracy, short course of treatment, and low toxicity and side effects.^[^ ^4^ ^]^ Researchers have carried out many clinical trials of BNCT technology in malignant tumors such as glioma, melanoma, head and neck tumors, and liver cancer, and have achieved initial results.^[^ ^5^ ^]^

The effective action of BNCT on malignant tumors depends on the targeted delivery of sufficient boron‐10 (^10^B) drugs to the tumor tissue to match the thermal neutrons that reach the tumor tissue at the proper moment and dose.^[^ ^6^ ^]^ The inadequate tumor uptake of all boron delivery agents currently used in clinical practice restricts the application of BNCT.^[^ ^7^ ^]^ The development of novel boron delivery agents with high selectivity, easy delivery, and high boron payload is an area of active research.^[^ ^8^ ^]^

Nanoparticle drug delivery systems significantly increase the accumulation of drugs in the tumor site to significantly improve the efficacy.^[^ ^9^ ^]^ The most commonly used nanocarriers are liposomes, polymers, micelles, and carbon‐based materials.^[^ ^10^ ^]^ Carbon dots (CDs), a novel type of carbon‐based nanomaterials, offer favorable conditions for the construction of nanomedicines due to their diversified physical and chemical properties, good biocompatibility, distinctive optical qualities, and inexpensive cost.^[^ ^11^ ^]^ It has been reported that the nano‐delivery system constructed by exosomes and boron‐containing carbon dots can penetrate the blood‐brain barrier to target tumor tissues for BNCT of mouse models of glioma in situ.^[^ ^12^ ^]^ That paper innovatively used CD as the ^10^B agents for BNCT, demonstrating that CDs are feasible nanocarriers for BNCT drugs. Unfortunately, CDs do not have tumor‐targeting properties, and their ^10^B content and fluorescence wavelength can still be increased. If a CDs composite system with high ^10^B content, long wavelength fluorescence, and tumor targeting properties is successfully prepared, the composite system will have better application value in BNCT.

Human serum albumin (HSA), the most prevalent protein in plasma, serves as a reservoir and carrier for a variety of drugs due to its high water solubility, biocompatibility, exceptional ligand binding capacity, and tumor‐targeting capabilities.^[^ ^13^ ^]^ Although the exact mechanism underlying albumin accumulation in tumors is unclear, it has been demonstrated that albumin‐binding drugs have either passive targeting through enhanced permeability and retention (EPR) effects into tumors, or active targeting mediated by albumin‐binding proteins and receptors such as secreted protein acidic and rich in cysteine (SPARC).^[^ ^14^ ^]^ Albumin‐binding medicines have a promising clinical therapeutic transformation potential. The first nanotechnology‐based chemotherapeutic agent Abraxane (nab‐paclitaxel) is HSA‐bound paclitaxel.^[^ ^15^ ^]^ The success of nab‐paclitaxel shows the potential of albumin as a drug carrier for tumor therapy.

**Scheme 1:**
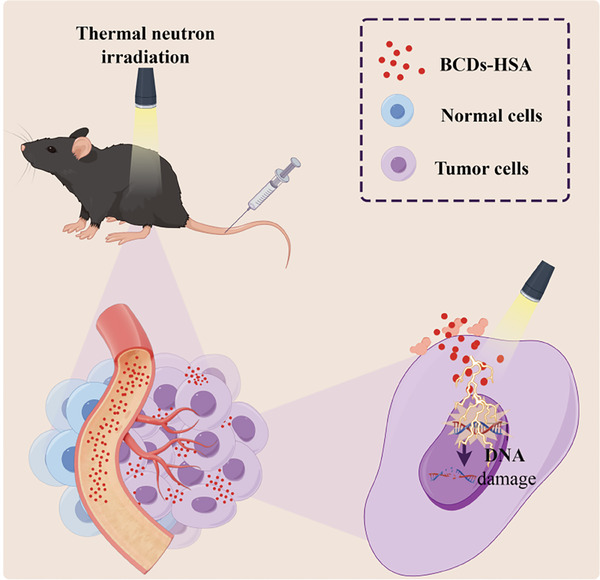
Schematic illustration of BCDs‐HSA for BNCT in a tumor mouse model. BCDs‐HSA targets tumor cells through EPR effect and SPARC, the DNA of tumor cells was damaged and apoptosis was induced under neutron irradiation. By Figdraw.

Here, we designed and synthesized novel boron‐containing carbon dots (BCDs) for BNCT in a mouse model of prostate cancer and a mouse model of melanoma (**Scheme** **1**). The prepared BCDs have a ^10^B content of approximately 7.1 wt%, with excitation wavelength‐independent orange emission and good biocompatibility. BCDs are the boron‐containing CDs with the longest fluorescence emission wavelength and the highest boron content for BNCT so far.^[^ ^12^, ^16^ ^]^ The inclusion of HSA increased cellular uptake and tumor accumulation of the complexes (BCDs‐HSA) significantly compared with only BCDs. In vitro cell cultures showed that the endocytosed BCDs‐HSA in cancer cells can significantly destroy cancer cells when exposed to thermal neutrons. Furthermore, using a prostate cancer mouse model and a melanoma mouse model, we discovered that BCDs‐HSA accumulated in tumors after intravenous treatment damaged the DNA of tumor cells, and significantly inhibited tumor growth when exposed to thermal neutron irradiation. This study proposes a high tumor‐targeting boron‐containing nanomedicine based on boron‐containing CDs and HSA, which could be used as a new type of boron‐containing medicine in BNCT.

## 2 Results and Discussion

### 2.1 Synthesis and Characterization of BCDs and BCDs‐HSA

**Figure 1:**
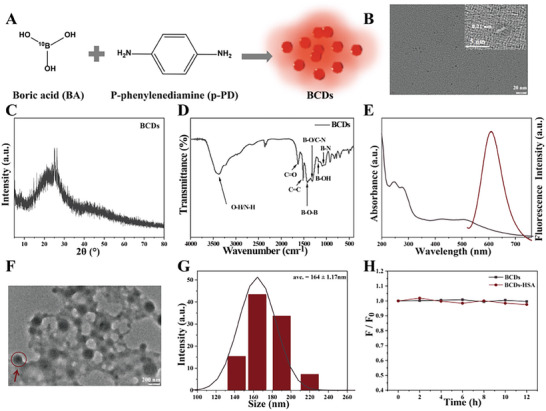
A) Schematic illustration of the synthetic process of BCDs. B) TEM image and HRTEM (the inset) image of BCDs. C) XRD pattern spectrum of BCDs. D) FT‐IR spectrum of BCDs. E) UV–vis absorption spectrum and fluorescence emission spectrum of BCDs. F) TEM image of BCDs‐HSA. G) Particle size distribution of BCDs‐HSA. H) Effect of placement time at 37 °C on fluorescence intensity of BCDs and BCDs‐HSA.

BCDs were synthesized by solvothermal method using boric acid and p‐phenylenediamine as raw materials (**Figure** **1**A).^[^ ^17^ ^]^ The morphology and size distribution of BCDs were investigated using transmission electron microscopy (TEM). As shown in Figure 1B, BCDs exhibit a uniform spherical morphology with a size range of 2.1–3.7 nm and an average diameter of 2.7 nm (Figure S1, Supporting Information). The lattice fringes of 0.21 nm corresponding to the (1 0 0) crystal plane of graphitic carbon were observed in the high‐resolution TEM (HRTEM) image of BCDs (illustrated in Figure 1B). The XRD pattern (Figure 1C) showed the crystalline structure of BCDs, with the typical peak center located at 2θ = 25.8°, corresponding to the (0 0 2) plane of graphite, which indicates that BCDs had both graphitic structure and amorphous carbon. The characteristic peaks of raw materials (BA and p‐PD) did not appear in the XRD pattern of BCDs, indicating that the raw materials in the BCDs had been decontaminated (Figure S2, Supporting Information). The chemical bonds and structures of BCDs were investigated by FT‐IR spectrum (Figure 1D; Figure S3, Supporting Information). The peaks at 3370, 1640, and 1520 cm^−1^ were separately attributed to the stretching vibrations of O─H/N─H, C═O, and C═C, respectively. The peaks at 1420, 1330, and 1180 cm^−1^ were assigned to the stretching vibration of B─O─B, B─O/C─N, and the bending vibration of B─OH, respectively. These results indicated that boric acid was successfully introduced into BCDs through chemical interaction. The weight content of ^10^B in BCDs was measured to be 7.1 wt% by ICP‐AES, which is higher than the ^10^B content of the clinical drug BPA of BNCT (5.2 wt%) and boron‐containing CDs that have been reported for BNCT (0.7805 wt% and 0.7524 wt%).^[^ ^12^, ^16^ ^]^

The optical properties of BCDs were further verified by UV‐Vis absorption spectroscopy and fluorescence emission spectroscopy. As shown in Figure 1E, BCDs exhibited two characteristic absorption peaks at 240 and 280 nm, which were derived from the π–π* transition of C═C and the n‐π* transition of C═O/C─N, respectively, indicating the presence of carbonyl and amino groups in BCDs. The broad absorption band of BCDs at 400–600 nm was due to the presence of many conjugated functional groups on the surface. The ─NH_2_, ─OH, and ─COOH groups on the surface of BCDs may be the reason for their excellent solubility in water. When the aqueous solution of BCDs was excited at 480 nm, the emission peak reached the maximum at 610 nm. While the excitation wavelength was in the range of 380–540 nm, the fluorescence emission peaks of BCDs showed a maxima shift of only <10 nm, which indicated that BCDs have an excitation‐independent fluorescence emission behavior (Figure S4, Supporting Information).

To solve the problem of rapid elimination caused by the ultrasmall particle sizes (2.7 ± 0.03 nm) of BCDs, we introduced HSA into BCDs to increase their retention time in tumors. Moreover, the tumor‐targeting properties of HSA allow better targeting of BCDs to increase their accumulation in the tumor site. The dual targeting effect would achieve a match between the boron dose in the tumor and the thermal neutron exposure time of BNCT duration. The complexes of HSA and BCDs (BCDs‐HSA) were obtained by blending BCDs and HSA in water at 40 °C for 1 h. Considering the easy aggregation characteristics of BCDs and moderate heat treatment, it can be inferred that BCDs‐HSA is a cluster formed by multiple BCDs entangled by HSA. TEM image showed that BCDs‐HSA was composed of HSA entangled in multiple BCDs (Figure 1F). The average hydrodynamic diameters of BCDs‐HSA were significantly enlarged to 164 ± 1.17 nm (Figure 1G). Circular dichroism spectroscopy analysis showed that the conformation of HSA in the complex was almost unchanged (Figure S5, Supporting Information). UV–vis absorption and fluorescence emission spectra showed that the optical properties of BCDs‐HSA were hardly affected (Figure S6, Supporting Information).

The good fluorescence stability presaged the potential application of BCDs and BCDs‐HSA in bioimaging fields. As shown in Figure 1H, the fluorescence intensity of the aqueous solution of BCDs and BCDs‐HSA remained virtually constant after being placed at 37 °C for 12 h, indicating that the fluorescence of the materials had good fluorescence stability. In addition, the fluorescence intensity of BCDs and BCDs‐HSA in 0 to 1.0 m NaCl solutions was negligible and unchanged (Figure S7, Supporting Information), indicating that their fluorescence was not affected by ionic strength.

### 2.2 Biocompatibility and BNCT Efficacy Evaluation In Vitro

**Figure 2:**
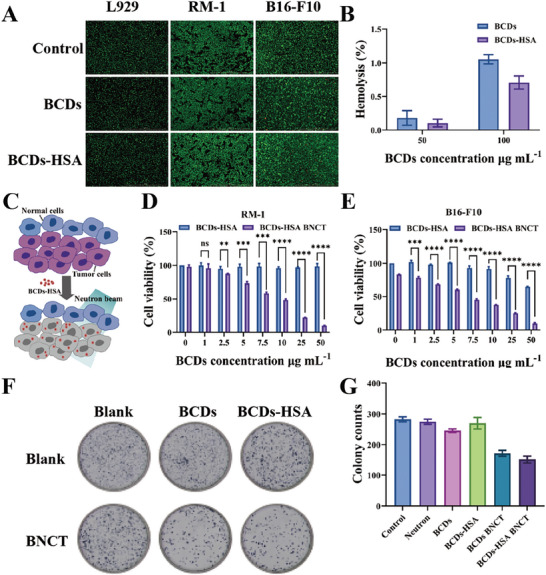
A) Live/dead viability assays were performed in L929 cells, RM‐1 cells, and B16‐F10 cells incubated with BCDs or BCDs‐HSA. B) The hemolysis rates of BCDs and BCDs‐HSA co‐incubated with mouse blood. C) Concept map of BNCT in cells treated with BCDs‐HSA. D) Cell viabilities of RM‐1 cells treated with BCDs‐HSA before and after neutron irradiation. E) Cell viabilities of B16‐F10 cells treated with BCDs‐HSA before and after neutron irradiation. F) Colony formations of RM‐1 cells with different drug groups before and after neutron irradiation. G) Number of RM‐1 cell colonies in the colony formation assay. Error bars denote standard errors (*n* = 3).

Good biocompatibility is the premise of the application of biomaterials. Cytotoxicity and hemocompatibility are important indicators. In this work, cell viability was measured by MTT assay. After L929 cells, RM‐1 cells, and B16‐F10 cells were incubated with different concentrations of BCDs or BCDs‐HSA for 24 h, it was found that the cell viability of different concentration groups was not affected (Figure S8, Supporting Information). And the introduction of HSA reduced the impact of BCDs on cell viability. After treatment with BCDs or BCDs‐HSA for 24 h, L929 cells, RM‐1 cells, and B16‐F10 cells were stained with calcein‐AM and PI. As shown in **Figure** **2**A, most cells were live cells emitting green fluorescence. And there was no significant difference among the different concentration groups (Figure S9, Supporting Information). The above results all indicated that BCDs and BCDs‐HSA did not show cytotoxicity. Subsequently, hemolysis testing was applied to evaluate the biosafety of BCDs and BCDs‐HSA. After co‐incubating fresh mouse whole blood with BCDs and BCDs‐HSA for 4 h, the erythrocytes remained well‐morphologized (Figure S10, Supporting Information), indicating that the drugs did not cause damage to cell morphology. In addition, hemolysis tests under static conditions were used to investigate the blood compatibility of materials. Diluted mouse blood was co‐incubated with deionized water, physiological saline, BCDs, and BCDs‐HSA for 1 h before centrifugation. It was found that except for the positive control group, there was no significant discoloration in the supernatant of BCDs or BCDs‐HSA, which was similar to that of the normal saline group (Figure S11, Supporting Information). Further determination of the supernatant revealed that the hemolysis rates of the experimental groups were all below 1.5%, and the hemolysis rates of the BCDs‐HSA groups were lower than those of the BCDs groups at the same concentration (Figure 2B). The hemolysis experiments illustrated that BCDs and BCDs‐HSA were well compatible with blood and would not affect blood cells, which provided a preliminary guarantee for subsequent animal experiments. The above experimental results demonstrated that both BCDs and BCDs‐HSA have good biocompatibility, and the introduction of HSA makes BCDs have better biosafety.

The BNCT efficacy in vitro of BCDs and BCDs‐HSA was investigated to verify their feasibility as excellent BNCT drugs (Figure 2C). The effects of BCDs or BCDs‐HSA on cell viability under neutron irradiation or without irradiation were evaluated by MTT assay, after mixed with RM‐1 cells or B16‐F10 cells for 12 h. Both BCDs and BCDs‐HSA showed good cell compatibility without neutron irradiation, but showed tumor cell cytotoxicity with increasing concentration under neutron irradiation (Figure 2D,E; Figure S12, Supporting Information). As shown in Figure S13 (Supporting Information), the half maximal inhibitory concentration (IC_50_) of the BCDs‐HSA BNCT group was lower than that of the BCDs BNCT group in both RM‐1 cells and B16‐F10 cells. Interestingly, the IC_50_ of the BCDs‐HSA BNCT group in B16‐F10 cells was smaller than that in RM‐1 cells (6.139 µg mL^−1^ < 9.817 µg mL^−1^), indicating that the BCDs‐HSA treated with neutron irradiation showed higher efficiency in killing B16‐F10 cells. The possible reason for this phenomenon was explored and discussed later. Variations in cell proliferative activity before and after neutron irradiation were investigated by clone formation assays. The number of RM‐1 cell colonies formed in the BCDs BNCT and BCDs‐HSA BNCT groups was significantly lower than that of the unirradiated BCDs and BCDs‐HSA groups. Furthermore, the cell colony number in the BCDs‐HSA BNCT group was fewer than that in the BCDs BNCT group (Figure 2F,G). Similarly, the number of B16‐F10 cell colonies formed in the BCDs‐HSA BNCT group was significantly lower than that of controls (Figure S14, Supporting Information). Finally, cells were stained with Annexin V‐FITC and PI to investigate the apoptotic status in RM‐1 cells. Compared with those no irradiation groups, BCDs or BCDs‐HSA combined with BNCT treatment significantly increased the apoptosis rate of RM‐1 cells (from 4.88% to 17.77% for BCDs, from 3.48% to 21.86% for BCDs‐HSA, respectively) (Figure S15, Supporting Information).

### 2.3 Exploration of Tumor Targeting and Accumulation

**Figure 3:**
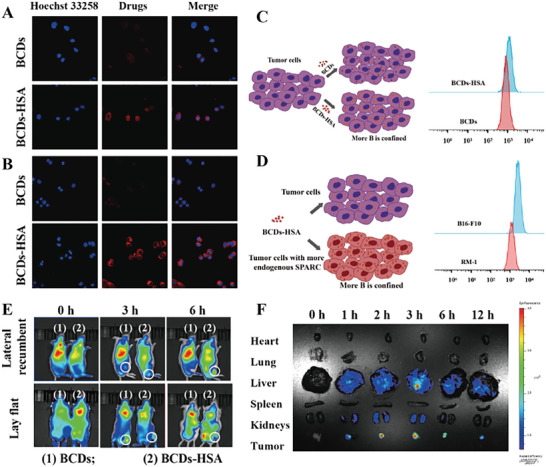
A) Cellular uptake images of BCDs and BCDs‐HSA by RM‐1 cells after 1 h of incubation. B) Cellular uptake images of BCDs and BCDs‐HSA by B16‐F10 cells after 1 h of incubation. C) Concept map of passive targeting effect of BCDs‐HSA (left), and cellular uptake of BCDs or BCDs‐HSA evaluated by flow cytometric quantitative analysis in RM‐1 cells when treated with BCDs or BCDs‐HSA for 0.5 h (right). D) Concept map of active targeting effect of BCDs‐HSA (left), cellular uptake of BCDs‐HSA evaluated by flow cytometric quantitative analysis in RM‐1 cells or B16‐F10 cells when treated with BCDs‐HSA for 0.5 h (right). E) In vivo fluorescence images of mice bearing RM‐1 tumors after intravenous injection of BCDs and BCDs‐HSA. F) Ex vivo fluorescence images of the tumors and organs harvested from the mice bearing RM‐1 tumors at different time points post‐injection of BCDs‐HSA.

The results of biocompatibility experiments and BNCT effect evaluations in vitro verified that BCDs and BCDs‐HSA could be applied as potential drugs for BNCT in tumor therapy. Then, the cellular uptakes of BCDs and BCDs‐HSA were thoroughly investigated. Flow cytometry results showed that both BCDs and BCDs‐HSA had time‐dependent cellular uptake behavior and could be taken up by cells within 0.5 h (Figure S16, Supporting Information). The fluorescence images of cells treated with BCDs or BCDs‐HSA for 0.5 or 1 h also confirmed the above viewpoint (**Figure** **3**A,B; Figure S17, Supporting Information).

It has been reported that the accumulation of HSA in tumors is due to the passive tumor‐targeting by the EPR effect and the active targeting mediated by secreted protein acidic and rich in cysteine (SPARC).^[^ ^14^, ^18^ ^]^ In our work, RM‐1 cells (prostate cancer cells) were selected as the representative of tumor cells with low expression levels of endogenous SPARC, and B16‐F10 cells (melanoma cells) were chosen as the representative of tumor cells with high expression level of endogenous SPARC.^[^ ^19^ ^]^ The impact of the introduction of HSA on the targeting ability of BCDs was explored. BCDs or BCDs‐HSA with the same ^10^B content were co‐incubated with RM‐1 cells for 0.5, 1, 2, and 4 h, then subjected to flow cytometry detection. The results showed that the uptake of BCDs‐HSA by RM‐1 cells was higher than that of pure BCDs at the same time point (Figure 3C; Figure S18, Supporting Information). Subsequently, when BCDs‐HSA was co‐incubated with RM‐1 cells or B16‐F10 cells, it was found that B16‐F10 cells could uptake more amount of BCDs‐HSA than that of RM‐1 cells (Figure 3D; Figure S19, Supporting Information), showing clear evidence of SPARC related targeting effect for BCDs‐HSA. The boron content in cells was measured by ICP‐AES after co‐incubation of BCDs or BCDs‐HSA with cells for 4 h. The concentration of ^10^B in RM‐1 cells co‐incubated with BCDs was 8.36 ± 1.24 µg/10^5^ cells, while the concentration of ^10^B in RM‐1 cells co‐incubated with BCDs‐HSA was 9.26 ± 0.53 µg/10^5^ cells. The amount of ^10^B in B16‐F10 cells co‐incubated with BCDs was calculated to be 7.95 ± 1.69 µg/10^5^ cells, while in B16‐F10 cells co‐incubated with BCDs‐HSA was 12.79 ± 1.88 µg/10^5^ cells. The above results illustrated that the introduction of HSA can enhance the accumulation of BCDs in tumor cells, providing a dual‐targeting function of active and passive targeting for BCDs‐HSA. The enhanced tumor‐targeting behavior of BCDs‐HSA was responsible for not only the better in vitro BNCT efficacy compared to BCDs, but also the better BNCT effect of BCDs‐HSA in B16‐F10 cells in comparison with RM‐1 cells.

BCDs and BCDs‐HSA were further administrated to tumor‐bearing mice to evaluate their in vivo biodistribution and tumor accumulation using fluorescence imaging and ICP‐AES. After intravenous injection of BCDs and BCDs‐HSA with the same boron content, in vivo and ex vivo fluorescence images of major organs (heart, liver, spleen, lung, and kidney) and tumors at different time points were performed on mice that bearing subcutaneous RM‐1 cells on the thigh. As shown in Figure 3E, the accumulation of BCDs‐HSA in the tumor gradually increased with time and maintained for >6 h. However, the accumulation of BCDs in the tumor was very limited and gradually disappeared from the tumor between 3 and 6 h, indicating that BCDs were not only less accumulated in the tumor, but also easier to be cleared. In another experiment, after intravenous injection of BCDs or BCDs‐HSA, the major organs and tumors were excised at different time points and studied for ex vivo fluorescence imaging. The obvious fluorescence signals in the BCDs group and BCDs‐HSA group can be observed in tumors, liver, and kidneys, because the liver is the main metabolic organ of drugs, and tumors are the main target sites for drug accumulation. For the BCDs group, the fluorescence intensity in the tumors reached a maximum at 2 h post‐injection and disappeared at 6 h post‐injection (Figure S20, Supporting Information). While in the case of the BCDs‐HSA group, the fluorescence intensity in the tumors reached a maximum at 3 h post‐injection and still remained obviously even after 12 h post‐injection (Figure 3F). The extended retention time of BCDs‐HSA in tumors was beneficial for matching the boron concentration in tumors that are required for effective BNCT. The boron content in tumors and normal organs was further measured using ICP‐AES for the mice bearing subcutaneous RM‐1 cells and B16‐F10 cells at 3 h post‐injection. The ^10^B concentrations in the dissected tumor tissues were calculated to be 28.19 ± 0.86 µg g^−1^ and 33.47 ± 2.14 µg g^−1^ for RM‐1 tumors and B16‐F10 tumors, respectively, well meeting the requirements to carry out BNCT, which means that the ^10^B content in the tumor is at least 20 µg g^−1^. The ratios of ^10^B concentration in the tumor to that in the surrounding normal tissue (T/N ratios) of RM‐1 tumor‐bearing mice treated with BCDs or BCDs‐HSA were 2.96 ± 0.24 and 5.94 ± 0.82, respectively. While the T/N ratios of B16‐F10 tumor‐bearing mice treated with BCDs or BCDs‐HSA were 3.02 ± 0.95 and 6.53 ± 0.51. The T/N ratios of tumor‐bearing mice treated with BCDs‐HSA also met the requirement of BNCT with a T/N ratio >3. At the same time, the T/N ratios after injection of BCDs and BCD‐HSA were higher than those of the clinical drug BPA of BNCT (2.03 ± 0.08) and the corresponding T/N ratios that have been reported for BNCT (BCDs: 2.91 ± 0.13 and BCDs‐Exos: 5.28 ± 0.29).^[^ ^12^ ^]^

### 2.4 BNCT in a Mouse Model of Prostate Cancer

**Figure 4:**
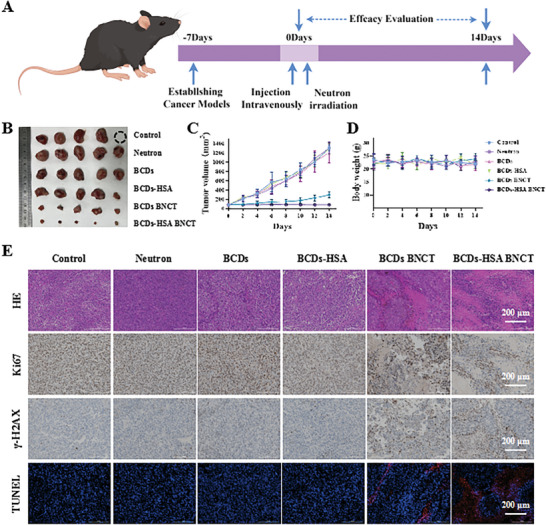
A) Scheme of experimental timeline. By Figdraw. B) Image of RM‐1 tumors isolated from mice. C) Tumor growth inhibition curves. D) Body weight changes of the mice over 14 days. E) Representative pathological images of RM‐1 tumor tissues of HE, Ki67, γ‐H2AX, and TUNEL staining.

In order to verify the BNCT effect of BCDs‐HSA in vivo, RM‐1 subcutaneous tumor models were established. Mice were randomly divided into six groups: BCDs, neutron‐irradiated BCDs (BCDs BNCT), BCDs‐HSA, neutron‐irradiated BCDs‐HSA (BCDs‐HSA BNCT), neutron and PBS as control groups (*n* = 5). The neutron irradiation was introduced at 3 h after injection. All mice survived to the end of the experiment (14 days) except one mouse in the PBS group died on the 7th day after the start of treatment (Figure S21, Supporting Information). It was shown that no clear tumor inhibition was confirmed in the control, neutron, BCDs, and BCDs‐HSA groups, while significant tumor inhibition was found in the BCDs BNCT and the BCDs‐HSA BNCT groups (**Figure** **4**B,C; Figure S22A, Supporting Information). We present tumor inhibition more intuitively by calculating the percentage change in tumor volume of each group of drugs in each mouse before and after treatment (tumor suppression rate). Tumor suppression rate = (tumor volume before treatment – tumor volume after treatment)/tumor volume before treatment × 100%. A positive tumor suppression rate means that the tumor is being suppressed and a higher positive number means that the tumor is being suppressed more effectively, while a negative tumor suppression rate indicates a promoting effect on tumor growth. Among these, the BCDs‐HSA BNCT group showed the best tumor suppression rate of ≈11.00% with completely depressed tumor growth (Figure S22B, Supporting Information). This increased BNCT efficacy was attributed to greater intra‐tumor ^10^B accumulation due to the passive targeting of BCDs‐HSA, which corroborated with the cellular assay results. There were no significant changes in the body weights of the mice in each group during the treatment (Figure 4D). What's more, white blood cell (WBC), red blood cell (RBC), hemoglobin (HGB), hematocrit (HCT), mean corpuscular volume (MCV), mean corpuscular hemoglobin (MCH), mean corpuscular hemoglobin concentration (MCHC), platelet (PLT) in blood and alanine transaminase (ALT), aspartate transaminase (AST), urea nitrogen (UREA), and creatinine (CREA) in serum were detected in all groups of mice at 14 days after treatment. The blood routine indexes of mice were in the normal range and there was no significant difference among the groups, which indicated that the mice in each group were healthy (Figure S23, Supporting Information). The ALT and AST values in the serum of the mice were in the normal range and there was no significant difference among the groups, indicating that the liver function of the mice in each group was normal (Figure S24, Supporting Information). The values of UREA and CREA in the serum of mice were in the normal range and there was no significant difference among the groups, indicating that the renal function of mice in each group was normal (Figure S24, Supporting Information). Major organs such as the heart, liver, spleen, lungs, and kidneys were collected and stained with hematoxylin and eosin (HE), and no histological abnormalities were observed in any group (Figure S25, Supporting Information). The above results indicated that BCDs‐HSA has a good BNCT effect in vivo without toxic and side effects.

To further investigate the mechanism, we carried out a pathology analysis of tumor tissues ex vivo (Figure 4E). HE staining showed that in the BCDs BNCT and BCDs‐HSA BNCT groups, the nuclei of cells showed indistinct borders and fragmentation. While in other groups, tumor cells had normal morphology and a high percentage of Ki67‐positive cells. The decrease of Ki67‐positive cells in the BNCT group indicated that BNCT reduced the activity of tumor proliferation. γ‐H2AX is a good protein marker for DNA damage. Immunohistochemical analysis of γ‐H2AX showed that the signal intensity of γ‐H2AX in BCDs‐HSA BNCT and BCDs BNCT groups was much higher than that of other groups, which proved that the boron drugs caused DNA damage in tumor cells under neutron irradiation. TUNEL detection is a commonly used method for detecting DNA damage based on the products formed after DNA damage. TUNEL analysis showed that the BCDs BNCT and BCDs‐HSA BNCT groups had more pronounced fluorescence signals compared to other groups. The results of TUNEL testing also confirm that the boron drugs caused DNA damage to tumor cells under neutron irradiation. The above experimental results indicated that under neutron irradiation, BCDs and BCDs‐HSA could efficiently induce tumor cell apoptosis by damaging DNA.

### 2.5 BNCT in a Mouse Model of Melanoma

**Figure 5:**
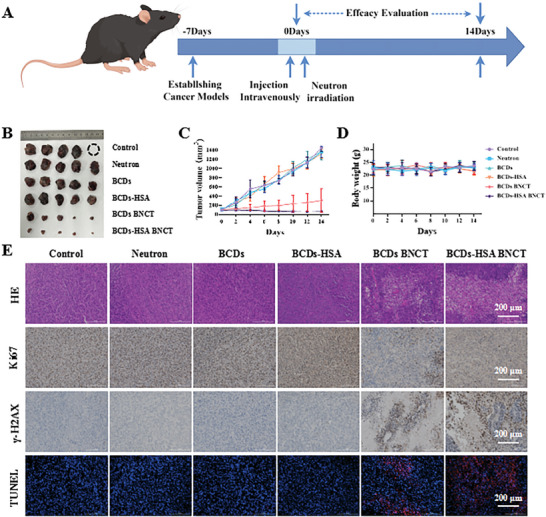
A) Scheme of experimental timeline. By Figdraw. B) Image of B16‐F10 tumors isolated from mice. C) Tumor growth inhibition curves. D) Body weight changes of the mice over 14 days. E) Representative pathological images of B16‐F10 tumor tissues of HE, Ki67, γ‐H2AX, and TUNEL staining.

To better verify the in vivo BNCT performance of BCDs‐HSA and the effect of its targeting on tumor suppression, the B16‐F10 subcutaneous tumor model was established. Similarly, the mice were randomly divided into six groups (*n* = 5) and administered intravenously. The neutron irradiation was introduced at 3 h after injection for BCDs BNCT and BCDs‐HSA BNCT groups. All mice survived to the end of the experiment (14 days) except one mouse in the PBS group died on the 5th day after the start of treatment (Figure S26, Supporting Information). Similar to the results in the above prostate cancer model, the BCDs‐HSA BNCT group showed the best tumor suppression effect in the mouse model of melanoma (**Figure** **5**B,C; Figure S27A, Supporting Information). The BCDs‐HSA BNCT group showed a higher tumor suppression rate in the mouse melanoma model than in the mouse prostate cancer model (29.35% > 11.00%), which was associated with more intra‐tumor ^10^B accumulation due to the active targeting of BCDs‐HSA (Figure S27B). The body weights of mice in each group did not change significantly during the treatment (Figure 5D). There was no significant difference in the blood biochemical indexes of the mice in each group after treatment and they were all within the normal range (Figures S28 and S29, Supporting Information). The analysis of HE staining results of the main organs of mice showed that no abnormalities were observed in the treatment groups compared with the control (Figure S30, Supporting Information). Similarly, HE, Ki67, γ‐H2AX, and TUNEL staining were used for further pathological analysis of ex vivo B16‐F10 tumor tissues. As shown in Figure 5E, the BCDs‐HSA BNCT group showed more DNA damage and apoptosis for B16‐F10 subcutaneous tumor.

## 3 Conclusion 

In summary, we reported the boron‐containing and HSA‐coated carbon dots BCDs‐HSA as the novel potential BNCT reagent. Compared with previous reports, the significant advantageous features of BCDs‐HSA are: 1) higher ^10^B content; 2) excellent tumor targeting ability and extended retention time provided by HSA coatings and 3) excitation‐independent fluorescent emission to support the application of fluorescence imaging for^10^B tracking in vivo. These characteristics of BCDs‐HSA enhanced the accumulation of ^10^B in tumors, thereby improving the effectiveness of BNCT. The characterization, biocompatibility, tumor targeting, and BNCT effects of BCDs‐HSA were explored in detail. The significant inhibitory effect of BCDs‐HSA on subcutaneous RM‐1 and B16‐F10 tumors under neutron irradiation also demonstrated the potential of BCDs‐HSA for BNCT on different cancers. To our knowledge, BCDs are currently the only CDs materials used for BNCT that combine extremely high ^10^B content and long wavelength fluorescence. We have effectively integrated the three properties of high ^10^B content, good tumor targeting, and fluorescence imaging, enabling BCDs‐HSA to have the function of “one stone, three birds”. The successful synthesis and excellent properties of BCDs‐HSA provide a feasible idea for the design of targeted boron‐containing CDs materials with tracer function, which has good inspiration and application value. In addition to that, the excellent optical properties of BCDs make them have the potential to be used in photothermal/photodynamic therapy, which can provide more possibilities for the combined treatment of BNCT.

## 4 Experimental Sections

### Reagents and Apparatus

P‐phenylenediamine (p‐PD, 99%) and boric acid (BA, ≥ 99.8%) were purchased from Shanghai Macklin Biochemical Co., Ltd.^10^BA (^10^B ≥ 95%) was purchased from Dalian Boronten Sci&Tech Co., Ltd. All reagents were used directly without further purification. The JEM‐2100F field emission transmission electron microscope was used to characterize the morphology of samples. The powder XRD patterns of samples were obtained by Smartlab X‐ray powder diffractometer. The infrared spectra of samples were obtained by Nicolet IS50 FT‐IR spectrophotometer. The F‐4600 fluorescence spectrometer (Hitachi, Japan) was used to detect the excitation and emission wavelengths of samples. The absorption spectra of samples were recorded by a Cary 50 UV–vis spectrophotometer. Zetasizer Nano ZSE was used to characterize particle size and polydispersity of samples. Circular dichroism (CD) was obtained by JASCO J‐500 circular dichroism. The boron content of the samples was analyzed by an inductively coupled plasma emission spectrometer (ICP‐AES, Prodigy). Flow cytometry analysis was performed using a Luminex flow cytometer (Amnis FlowSight). A fluorescence microplate reader (BioTek Cytation5) was used to capture cell fluorescence images and tissue section images. Laser Scanning Confocal Microscope (Olympus, FV‐3000) was used to observe cell fluorescence images. CRI Maertro 500FL was used for animal tissue imaging.

### Neutron Source

The NT503 neutron tube, ^3^He neutron monitor, and multi‐channel analyzer were provided by Northeast Normal University. The neutron generator produces fast neutrons, which can be slowed down into thermal neutrons using lead and high‐density polyethylene materials. The theoretical output of the neutron source is 5.4 × 10^9^ n s^−1^, with a neutron emission distance of 9.9 cm from the target body and a radius of 0.55 cm on the exit circular surface. The Monte Carlo simulation (MCNP5) is as follows:

(1) $$H=\frac{\phi_{i}\times Y\times T\times S}{K_{i}}$$

In the formula, H is the dose at the tumor site, ϕ_*i*_ is the neutron flux in different energy ranges, *Y* is the neutron yield, *T* is the irradiation time, *S* is the tumor area (radius = 0.55 cm), and *K*_i_ is the flux dose conversion coefficient in different energy ranges.

### Synthesis of BCDs and HSA Trapped with BCDs (BCDs‐HSA)

BCDs were synthesized directly by the one‐pot solvothermal method recorded in the literature.^[^ ^20^ ^]^ In short, p‐PD (1 mmol) and BA (5 mmol) were dissolved in 20 mL ethanol and sonicated for 10 min to mix evenly. The solution was then transferred into a stainless steel autoclave lined with polytetrafluoroethylene (50 mL) and heated at 200 °C for 10 h. After natural cooling to room temperature (RT), the obtained solution was filtered using a 0.22 µm filter to remove insoluble precipitates. The reddish‐brown solution was obtained and then dialyzed against deionized water for 24 h using a 1000 MWCO dialysis membrane. After vacuum drying at 60 °C the powder of BCDs was obtained. Then, 1 mg of BCDs was dissolved in 20 mg mL^−1^ HSA solution for 1 h under 40 °C to prepare HSA trapped with BCDs (BCDs‐HSA).

### Potential Cytotoxicity

Cytotoxicity profiles of BCDs and BCDs‐HSA against L929 cells, RM‐1 cells, and B16‐F10 cells were evaluated by the MTT assay. The cells were seeded on a 96‐well plate (3000–6000 cells well^−1^) and cultured overnight in an incubator. The drugs were added to the 96‐well plate after gradient dilution and incubated for 24 h. After that, MTT was added to the 96‐well plates, and incubation was continued for 4 h. After the medium was removed, DMSO was added to dissolve the blue‐purple crystals formed in the cells, which were shaken in a microplate reader for 3 min to fully dissolve, and then the absorbance OD value of each well at 490 nm was detected. Each experiment was performed three times for each cell line.

Cytotoxicity profiles of the drugs were observed using a Live/Dead cell double stain kit. Cells were inoculated in a 48‐well plate and then placed in an incubator for overnight incubation. Then the drugs were added to the 48‐well plate and incubated in the incubator for 24 h. Then Calcein‐AM and propidium iodide (PI) were added and cultured for 15 min. The labeled cells were visualized images were captured using a fluorescence microplate reader. Live cells were stained green, while the dead cells were stained red.

### Cell Uptake and Targeted Examination

RM‐1 cells or B16‐F10 cells were seeded in a 6‐well plate overnight culture. Medium containing BCDs or BCDs‐HSA (10 or 210 µg mL^−1^, respectively, equivalent ^10^B 0.712 µg mL^−1^) was added to the six‐well plate and incubated for different periods of time (0.5, 1, 2, and 4 h). After being washed three times with cold PBS, cells were collected and analyzed by flow cytometry. In addition, images of cellular endocytosis of drugs were captured by Laser Scanning Confocal Microscope. Boron analysis was carried out by ICP‐AES.

### Efficacy Assessments of BNCT on Cells

Cell activity was detected by MTT assay, which was divided into before and after neutron irradiation. The cells (3000 cells/well) were inoculated in a 96‐well plate for overnight culture. The drugs were added to the 96‐well plate after gradient dilution. Three replicate wells were set for each concentration. Neutron irradiation (2 Gy) was applied to the irradiated group after 12 h of incubation, while the non‐irradiated group was not treated. The cells were cultured for 24 h after neutron irradiation and then cultured for 4 h after adding MTT. After the medium was discarded and DMSO was added, the 96‐well plate was shaken in a microplate reader for 3 min. The absorbance OD value of each well at 490 nm was detected, and the relative survival rate of the cells was calculated for in vitro efficacy evaluation.

Cell proliferation ability was detected by a cell cloning test. The cells (1000 cells per well) were inoculated in a 6‐well plate for overnight culture. The drugs were added to the 6‐well plate and incubated for 12 h. Then neutron irradiation (2 Gy) was performed on the irradiated group, and no treatment was performed on the non‐irradiated group. Cells were Giemsa stained after 7 days and clone formation efficiency was calculated.

The apoptosis of cells was detected. RM‐1 cells (1 × 10^5^ cells per well) were inoculated in a 6‐well plate for overnight culture. The drugs were added to the 6‐well plate and incubated for 12 h. Cells in the BNCT group were irradiated with neutron irradiation (2 Gy), and no treatment was performed on the non‐irradiated group. 24 h after the irradiation, cells were stained by Annexin V‐FITC/PI, and the apoptosis of cells was detected by flow cytometry.

### Hemolysis Test

The 5% red blood cell diluent was mixed with normal saline, deionized water, BCDs, and BCDs‐HSA at a volume ratio of 1:1, respectively. The mixture was incubated at 37 °C for 1 h and centrifuged at 1500 rpm for 5 min. The supernatant was taken out and placed in a 96‐well plate. The OD value of the supernatant at 540 nm was measured by a microplate reader and the percentages of hemolysis were calculated.

### Fluorescence Imaging and Biodistribution of Mouse Model

For fluorescence imaging, RM‐1 tumor‐bearing mice were administered with 10 mg kg^−1^ of BCDs or 210 mg kg^−1^ BCDs‐HSA through the tail vein. Then the mice were euthanized at 0, 1, 2, 3, 6, and 12 h, and tumors and viscera were taken out to achieve the fluorescence distribution in vitro. Boron analysis was carried out by ICP‐AES.

### Animal Models

RM‐1 cells were injected subcutaneously into the legs of male C57BL/6 (4 weeks, 15–20 g) mice to establish RM‐1 tumor models. B16‐F10 cells were injected subcutaneously into the legs of male C57BL/6 (4 weeks, 15–20 g) mice to establish B16‐F10 tumor models. BNCT experiments and T/N ratio tests were performed in RM‐1 tumor models and B16‐F10 tumor models. All animal experiments were approved and carried out according to the guidelines of the Ethical Committee of Northeast Normal University (Approval numbers: 202 402 042).

### In Vivo BNCT

Mice were randomly divided into 6 groups (PBS, BCDs, BCDs‐HSA, neutrons, BCDs BNCT, BCDs‐HSA BNCT) with 5 mice in each group when the tumor volume reached 100 mm^3^. The equivalent 210 mg kg^−1^ of ^10^B BCDs‐HSA and ^10^B BCDs with the same content of ^10^B were injected into mice via the tail vein. About 3 h after drug injection, neutron irradiation (2 Gy) was performed on the irradiated group, and the non‐irradiated group was not treated. In order to minimize the impact of radiation on normal tissues and organs, polyethylene protective devices are used during animal BNCT treatment.^[^ ^21^ ^]^ Neutrons will directly irradiate the tumor site of mice, while other body parts outside the small hole range are protected by protective devices. The weight and tumor volume of the mice were recorded every 2 days. At the end of the anti‐cancer experiment of 21 days, the mice were sacrificed and the tumors were isolated to obtain images. The main organs and tumor tissues were fixed with 4% paraformaldehyde buffer and then embedded in paraffin. The paraffin‐embedded tissue sections were examined by routine immunohistochemistry. Major organs were collected for HE analysis. Isolated tumors were further analyzed for HE, Ki67, γ‐H2AX, and TUNEL. All antibodies were purchased from Servicebio and all experimental procedures were carried out according to the instructions. Mouse blood was collected in anticoagulant tubes to obtain blood samples. Mouse blood was collected in procoagulant tubes and allowed to stand for 30 min, then centrifuged at 3000 r min^−1^ for 5 min to obtain serum samples. Blood and serum samples were analyzed using ABX micros60 and BS‐200 systems, respectively.

### Statistical Analysis

Experimental results were present as the mean ± SD. Student's *t*‐test was used for statistical significance. Statistical analysis between the two groups was noted as follows: ^*^ *p* < 0.05, ^**^ *p* < 0.01, ^***^ *p* < 0.001.

## Conflict of Interest

The authors declare no conflict of interest.

## Supporting information



Supporting Information

## Data Availability

The data that support the findings of this study are available from the corresponding author upon reasonable request.
