# Supplementary material for: Human Serum Albumin‐Coated 10B Enriched Carbon Dots as Targeted “Pilot Light” for Boron Neutron Capture Therapy
Source: Adv Sci (Weinh). 2024 Sep 26;11(43):2406577. doi: 10.1002/advs.202406577 (PMC11578295; doi:10.1002/advs.202406577)
Supplement: Supplementary file 1 — Supporting Information [file ADVS-11-2406577-s001.docx]

**Supporting Information**

**Human Serum Albumin-Coated ^10^B Enriched Carbon Dots as Targeted “Pilot Light” for Boron Neutron Capture Therapy**

Tianyuan Zhong^1^, Yongjin Yang^2, 3^, Miao Pang^1^, Yong Pan^1^, Shiwei Jing^4^, Yanxin Qi^1,*^, Yubin Huang^1, *^

*1 Faculty of Chemistry, Northeast Normal University, Changchun, 130024, China*

*2 Department of Urology, The second Hospital & Clinical Medical School, Lanzhou University, Lanzhou, 730000, Gansu, China.*

*3 Gansu Province Clinical Research Center for Urinary system disease, Lanzhou, 730000, Gansu, China.*

*4 School of Physics, Northeast Normal University, Changchun, 130024, China*


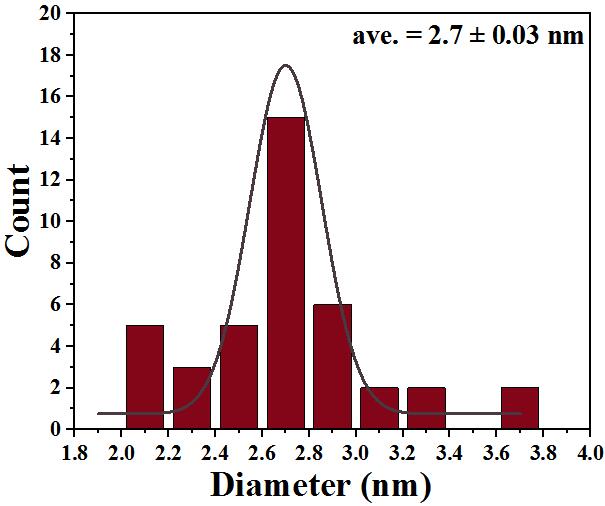


Fig. S1 Particle size distribution of BCDs.


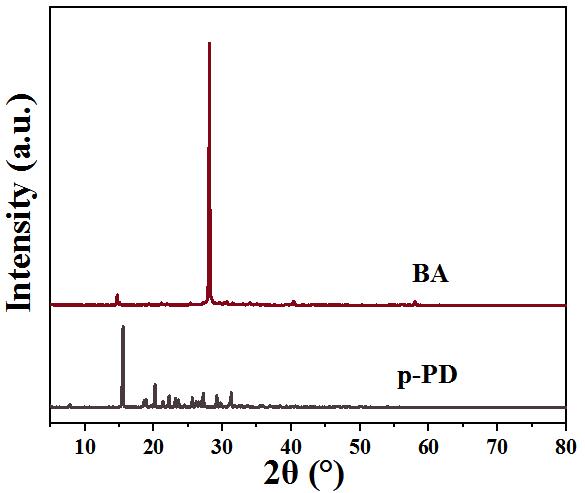


Fig. S2 XRD pattern spectra of BA and p-PD.


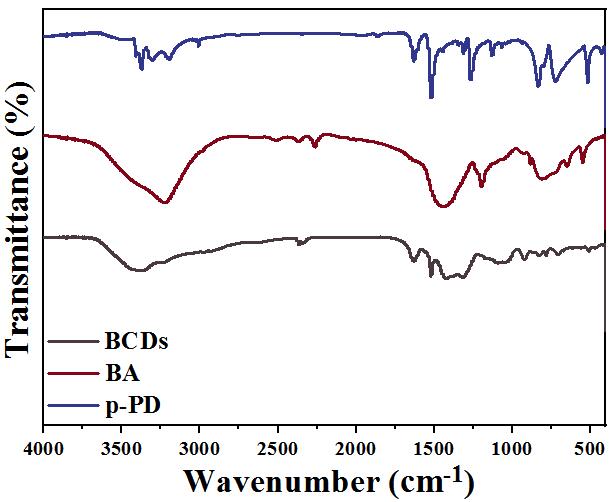


Fig. S3 FT-IR spectra of BCDs, BA and p-PD.


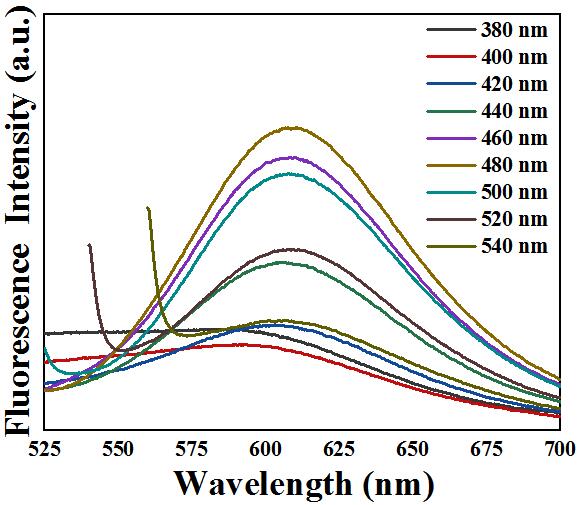


Fig. S4 Fluorescence emission spectra of BCDs with different excitation wavelengths from 380 to 540 nm.


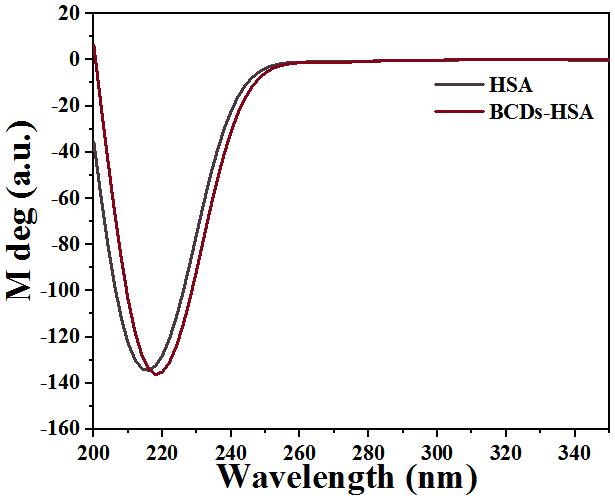


Fig. S5 Circular dichroism spectra of HSA and BCDs-HSA.


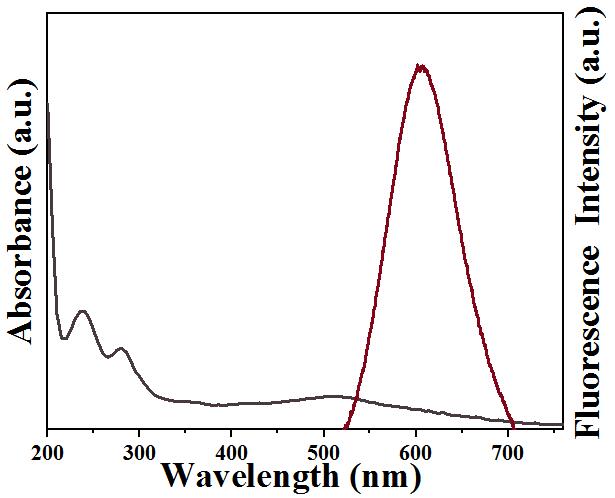


Fig. S6 UV-vis absorption spectrum and fluorescence emission spectrum of BCDs-HSA.


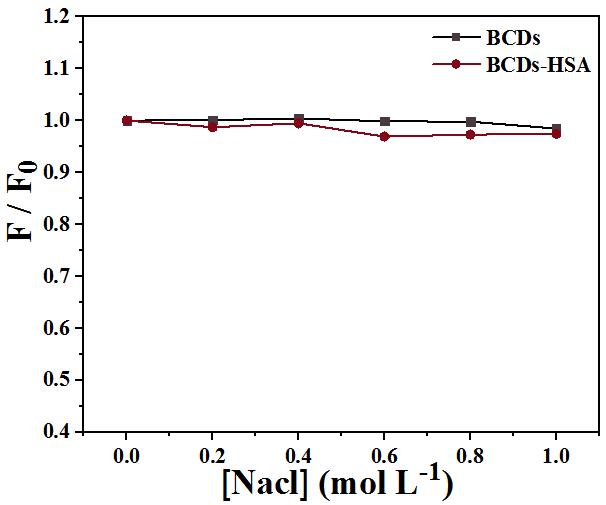


Fig. S7 Effect of NaCl concentration on the fluorescence intensity of BCDs and BCDs-HSA.


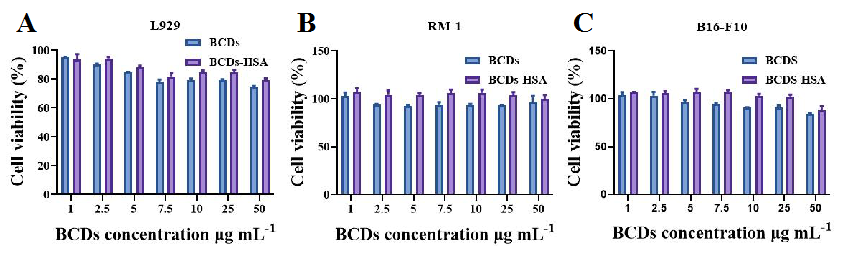


Fig. S8 Cell viabilities of A) L929 cells B) RM-1 cells and C) B16-F10 cells treated with BCDs or BCDs-HSA. Error bars denote standard errors (n = 3).


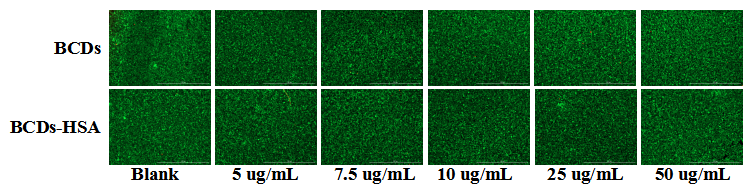


Fig. S9 Calcein-AM/PI images of RM-1 cells incubated with different concentrations of BCDs or BCDs-HSA.


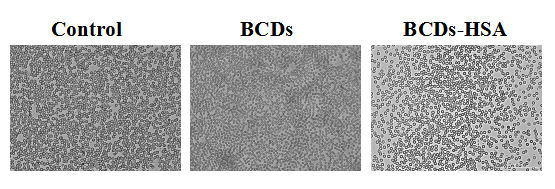


Fig. S10 Images of red blood cell morphology after co-incubation of BCDs or BCDs-HSA.


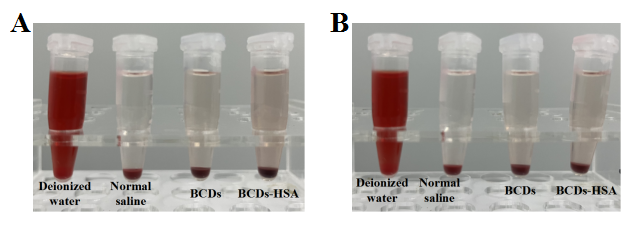


Fig. S11 Images of hemolysis tests with BCDs concentrations are A) 50 ug/mL and B) 100 ug/mL BCDs and BCDs-HSA.


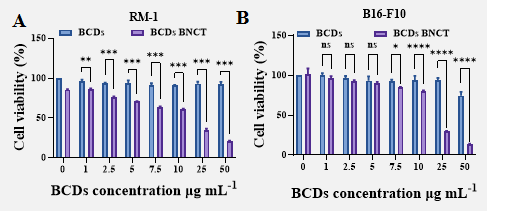


Fig. S12 Cell viabilities of A) RM-1 cells or B) B16-F10 cells treated with BCDs before and after neutron irradiation. Error bars denote standard errors (n = 3).


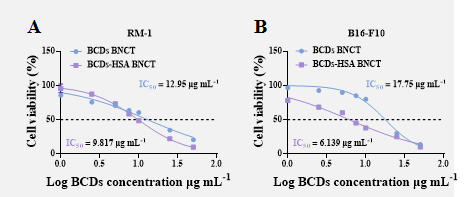


Fig. S13 IC_50_ of BCDs and BCDs-HSA under neutron irradiation in A) RM-1 cells and B) B16-F10 cells.


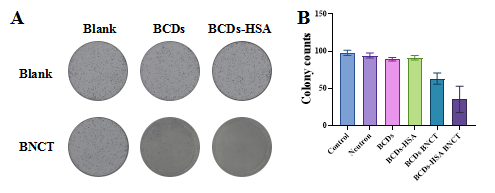


Fig. S14 A) Colony formations of B16-F10 cells with different drug groups before and after neutron irradiation; B) Number of B16-F10 cell colonies in the colony formation assay. Error bars denote standard errors (n = 3).


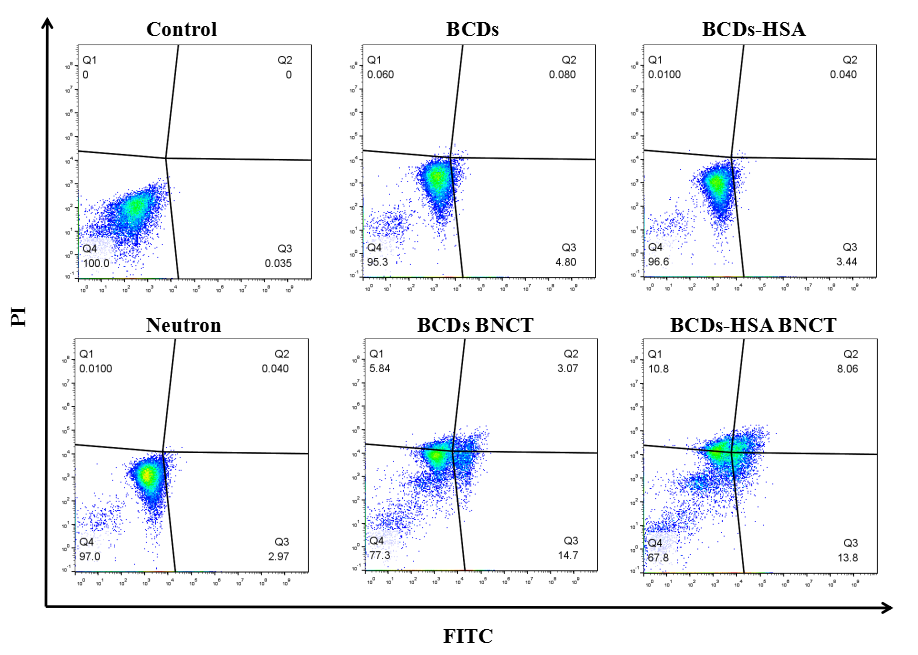


Fig. S15 Flow cytometric analysis and annexin V-FITC/PI staining to determine the apoptosis effect of RM-1 cells induced by BNCT.


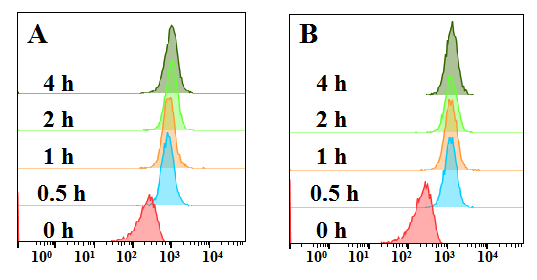


Fig. S16 Cellular uptake of A) BCDs or B)BCDs-HSA evaluated by flow cytometric quantitative analysis in RM-1 cells when treated with BCDs or BCDs-HSA for 0.5 h, 1 h, 2 h and 4 h.


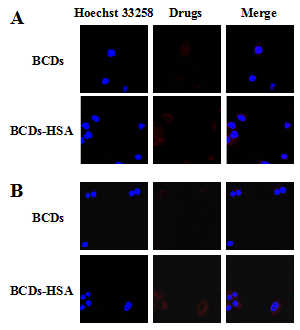


Fig. S17 A) Cellular uptake images of BCDs and BCDs-HSA by RM-1 cells after 0.5 h of incubation; B) Cellular uptake images of BCDs and BCDs-HSA by B16-F10 cells after 0.5 h of incubation.


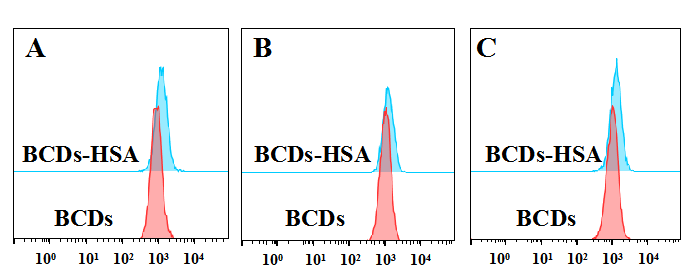


Fig. S18 Cellular uptake of BCDs or BCDs-HSA evaluated by flow cytometric quantitative analysis in RM-1 cells when treated with BCDs or BCDs-HSA for A) 1 h; B) 2 h; C) 4 h.


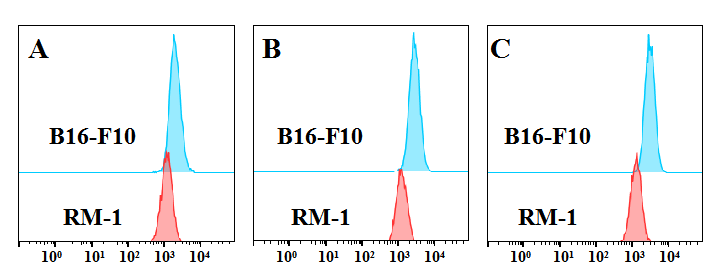


Fig. S19 Cellular uptake of BCDs-HSA evaluated by flow cytometric quantitative analysis in RM-1 cells or B16-F10 cells when treated with BCDs-HSA for A) 1 h; B) 2 h; C) 4 h.


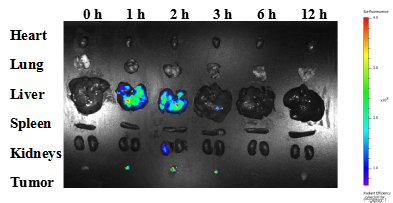


Fig. S20 *Ex vivo* fluorescence images of the tumors and organs harvested from the mice bearing RM-1 tumors at different time points post-injection of BCDs.


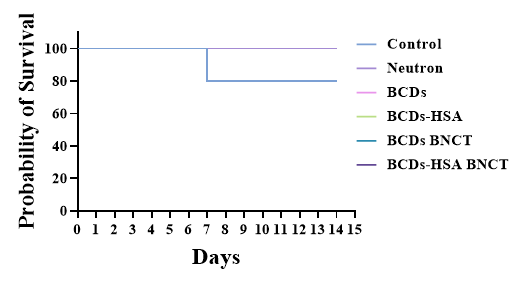


Fig. S21 Survival curves of RM-1 tumor-bearing mice after various treatments.


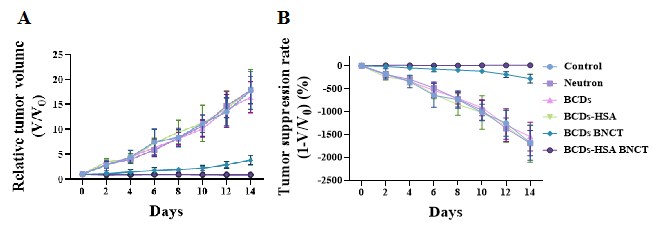


Fig. S22 A) Relative RM-1 tumor growth curves; B) RM-1 tumor suppression rate curves after various treatments.


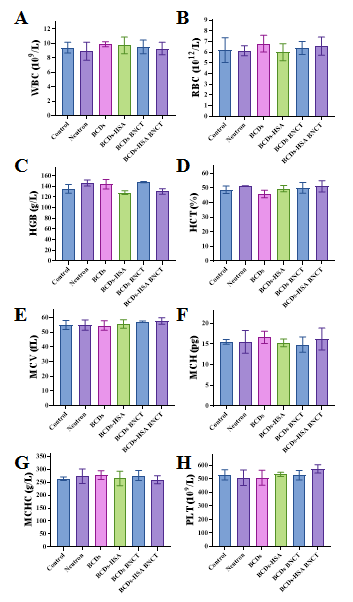


Fig. S23 Hematological date of RM-1 tumor-bearing mice after various treatments.


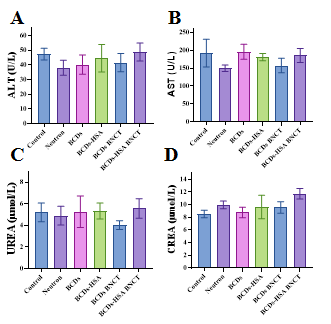


Fig. S24 Blood-biochemical analysis of RM-1 tumor-bearing mice after various treatments.


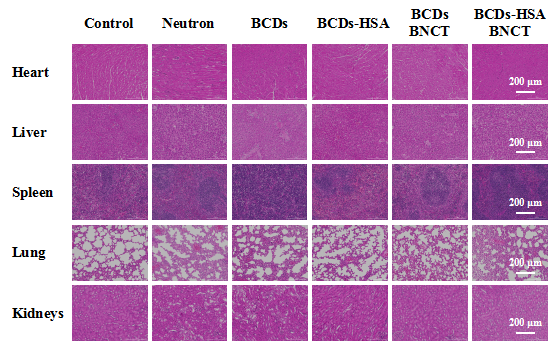


Fig. S25 Representative HE staining of major organs of RM-1 tumor-bearing mice after various treatments.


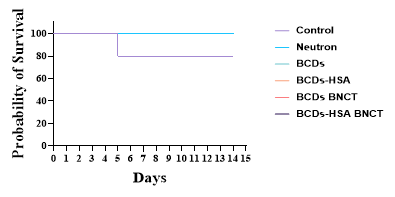


Fig. S26 Survival curves of B16-F10 tumor-bearing mice after various treatments.


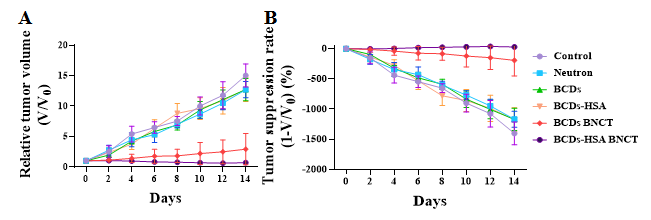


Fig. S27 A) Relative B16-F10 tumor growth curves; B) B16-F10 tumor suppression rate curves after various treatments.


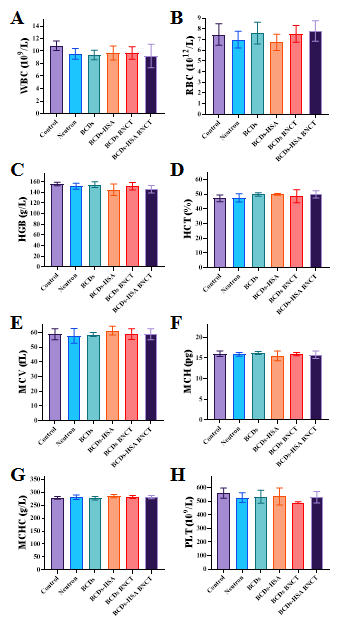


Fig. S28 Hematological date of B16-F10 tumor-bearing mice after various treatments.


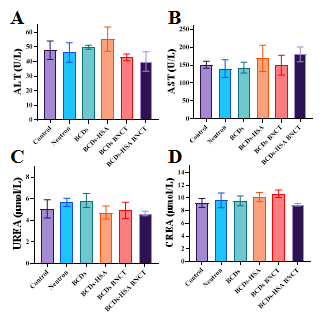


Fig. S29 Blood-biochemical analysis of B16-F10 tumor-bearing mice after various treatments.


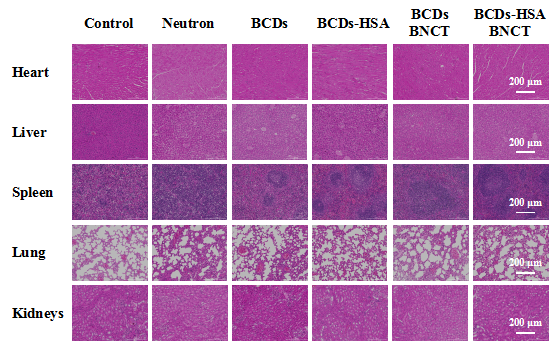


Fig. S30 Representative HE staining of major organs of B16-F10 tumor-bearing mice after various treatments.
